# Allosteric Remodelling of the Histone H3 Binding Pocket in the Pygo2 PHD Finger Triggered by Its Binding to the B9L/BCL9 Co-Factor

**DOI:** 10.1016/j.jmb.2010.07.007

**Published:** 2010-09-03

**Authors:** Thomas C.R. Miller, Trevor J. Rutherford, Christopher M. Johnson, Marc Fiedler, Mariann Bienz

**Affiliations:** MRC Laboratory of Molecular Biology, Hills Road, Cambridge CB2 0QH, UK

**Keywords:** Pygo, Pygopus, HD1, homology domain 1, T3, threonine 3, Lgs, Legless, ITC, isothermal titration calorimetry, HSQC, heteronuclear single-quantum correlation, wt, wild type, TEV, tobacco etch virus, Pygo, B9L/BCL9, methylated histone H3 tail, allosteric modulation, PHD signature residue

## Abstract

The Zn-coordinated PHD fingers of Pygopus (Pygo) proteins are critical for β-catenin-dependent transcriptional switches in normal and malignant tissues. They bind to methylated histone H3 tails, assisted by their BCL9 co-factors whose homology domain 1 (HD1) binds to the rear PHD surface. Although histone-binding residues are identical between the two human Pygo paralogs, we show here that Pygo2 complexes exhibit slightly higher binding affinities for methylated histone H3 tail peptides than Pygo1 complexes. We solved the crystal structure of the Pygo2 PHD–BCL9-2 HD1 complex, which revealed paralog-specific interactions in its PHD–HD1 interface that could contribute indirectly to its elevated affinity for the methylated histone H3 tail. Interestingly, using NMR spectroscopy, we discovered that HD1 binding to PHD triggers an allosteric communication with a conserved isoleucine residue that lines the binding channel for histone H3 threonine 3 (T3), the link between the two adjacent binding pockets accommodating histone H3 alanine 1 and methylated lysine 4, respectively. This modulates the surface of the T3 channel, providing a plausible explanation as to how BCL9 co-factors binding to Pygo PHD fingers impact indirectly on their histone binding affinity. Intriguingly, this allosteric modulation of the T3 channel is propagated through the PHD structural core by a highly conserved tryptophan, the signature residue defining the PHD subclass of Zn fingers, which suggests that other PHD proteins may also be assisted by co-factors in their decoding of modified histone H3 tails.

## Introduction

The Wnt/β-catenin signalling pathway controls a context-specific transcriptional programme that is essential for metazoan development and tissue homeostasis.^1,2^ Inappropriate activation of Wnt/β-catenin signalling can lead to cancer, especially colorectal cancer.^3,4^ The central effector of this pathway is β-catenin, which is constitutively degraded by the proteasome in the absence of Wnt but is stabilised upon recognition of a Wnt signal at the cell membrane. Once stabilised, β-catenin accumulates in the nucleus and associates with DNA-bound TCF/LEF factors, thus providing a platform for the recruitment of various general transcriptional co-activators to Wnt target genes, including chromatin modifiers and remodelling factors.^5^

Genetic screens in *Drosophila* led to the discovery of two additional factors that are essential for, and apparently dedicated to, the transcriptional activity of Armadillo (the *Drosophila* β-catenin) during normal development, namely, Pygopus (Pygo) and Legless (Lgs).^6–9^ Pygo contains a single zinc-coordinated PHD finger in its C-terminus, by which it binds directly to a short Lgs domain [called homology domain 1 (HD1)]; Lgs in turn uses its homology domain 2 to bind to Armadillo, thus forming a ‘chain-of-adaptors’.^7,10^ How Pygo and Lgs assist Armadillo as a transcriptional co-activator is unclear. Pygo could associate with Wnt target genes upon Wnt signalling, through the Lgs > Armadillo > TCF adaptor chain, to recruit an unknown transcriptional co-factor.^7,10^ However, more recently, it has been shown that Pygo can associate with Wnt target genes in the absence of Armadillo^11^ and may facilitate the efficient capture of nuclear Armadillo upon Wnt signalling.^12^ Notably, PHD_Pygo_–HD1_Lgs/BCL9_ complexes bind specifically to H3K4me (histone H3 tail methylated at lysine 4)^13,14^ like some other PHD fingers.^15–17^

Vertebrates have two orthologs of Pygo and Lgs: Pygo1 and Pygo2, and BCL9 and BCL9-2/B9L (referred to as B9L), which contribute to efficient β-catenin-dependent transcription in human colorectal cancer cell lines with high Wnt pathway activity.^9,18–20^ Overexpression of Pygo2, BCL9 and B9L has been reported for various human cancers,^18,21–23^ and BCL9 appears to have a role in promoting tumour progression.^24^ Knockout studies in mice revealed that both murine Pygo paralogs participate in β-catenin-dependent transcription in various tissues, although Pygo1 knockouts are viable, whereas Pygo2 knockouts are embryonic lethal.^14,25,26^ Likewise, knockout of Bcl9 paralogs revealed their role in β-catenin-dependent transcription and causes embryonic (Bcl9) or perinatal (Bcl9-2) lethality.^27^ In addition, these studies also revealed Wnt-independent functions of murine Pygo and Bcl9 paralogs (e.g., in eye lens development, spermiogenesis^28,29^ and adult skeletal muscle^27^), and BCL9 appears to have a lymphoid-specific transcriptional activation function.^30^ Interestingly, Pygo2 functions in mammary progenitor cells to bind to chromatin and to facilitate trimethylation of H3K4 at Wnt target genes and elsewhere.^14^ A picture of Pygo (and Bcl9) paralogs sharing redundant and overlapping functions is beginning to emerge, but they may also have paralog-specific roles in certain tissues and malignancies. Of the two Pygo paralogs, Pygo2 appears to be the more functionally relevant, both in normal development and in cancer.^14,21,22,25^

A number of different PHD fingers bind histone H3 tails through an anchoring pocket accommodating its N-terminal alanine 1 (A1) and a specificity pocket accommodating K4 that determines their preferences for specific K4 methylations (e.g., Taverna *et al.*,^17^ Li *et al.*,^31^ Wysocka *et al.*^32^). To our knowledge, Pygo PHD fingers are the only ones whose histone H3 tail binding is regulated by a co-factor ligand, namely, the HD1 of BCL9 proteins: human BCL9 HD1 significantly increases the affinity of human Pygo1 PHD for H3K4me, while Lgs HD1 is essential for H3K4me binding of *Drosophila* Pygo PHD.^13^ Surprisingly, the crystal structure of the ternary H3K4me2–PHD_Pygo1_–HD1_BCL9_ complex revealed that HD1 does not interact directly with the PHD histone pocket but binds to the opposite surface of the PHD finger, which forms the base of its A1 binding pocket. We thus proposed that HD1 buttresses this pocket, which is critical for anchoring the N-terminus of the histone H3 tail.^13^

Here, we explore the molecular mechanism by which mammalian HD1 assists PHD in binding to histone H3 tail. Given the emerging physiological differences between the murine Pygo paralogs and the murine Bcl9 paralog (see the text above), we measured the binding affinities of all four PHD and HD1 combinations for dimethylated histone H3 tail peptide (H3K4me2) by isothermal titration calorimetry (ITC) and found that the complex between Pygo2 PHD and B9L HD1 (PHD_Pygo2_–HD1_B9L_) has the highest affinity for H3K4me2. We thus solved the crystal structure of this complex, which closely resembles that of the PHD_Pygo1_–HD1_BCL9_ complex,^13^ but also revealed paralog-specific interactions in the PHD–HD1 interface. Using nuclear magnetic resonance (NMR) spectroscopy, we discovered a short-range allosteric reaction chain triggered by HD1 binding to PHD, targeting primarily a conserved isoleucine residue (I344) that lines the threonine 3 (T3) channel, the link between the two adjacent histone binding pockets accommodating histone H3 A1 and K4me. This allosteric communication is propagated through the PHD structural core by W377, which corresponds to the tryptophan signature residue that defines the PHD subclass of Zn fingers^33,34^ and is a key feature of PHD structural cores^35,36^ (note that this residue is distinct from W353, which separates the A1 pocket and the K4me pocket; see the text below). This raises the interesting possibility that other PHD fingers may also have co-factor ligands that modulate their histone H3 tail recognition.

## Results

### PHD–HD1 paralog complexes exhibit distinct affinities for methylated histone H3 tail peptides

**Fig. 1:**
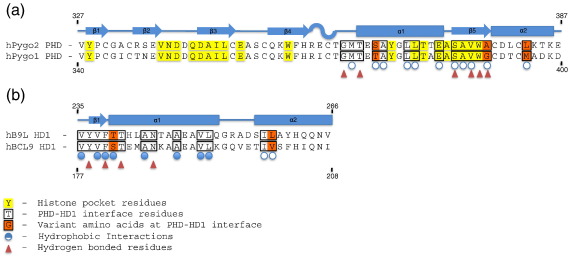
Structural elements and ligand-binding residues of human Pygo PHD and B9L HD1 paralogs. Sequence alignments of (a) PHD and (b) HD1 sequences, as indicated. Marked above the sequences are secondary structure elements (β-sheets and α-helices; α-turns marked by S shapes); indicated below are residues mediating PHD–HD1 interactions.

The PHD and HD1 of the Pygo and BCL9 paralogs are highly related to each other: there are 12 amino acid substitutions between the two PHD paralogs, all of which are solvent-exposed surface residues in the PHD_Pygo1_–HD1_BCL9_ complex;^13^ three of these are in the PHD–HD1-interacting surface, while the histone-pocket-lining residues are identical between the two Pygo paralogs (Fig. 1a). Similarly, the 14 amino acid substitutions between human BCL9 and B9L are all solvent exposed, except for BCL9 F203 (Y261 in B9L) which maintains the hydrophobic core of HD1, with only two substitutions in the HD1–PHD interface (Fig. 1b).

**Fig. 2:**
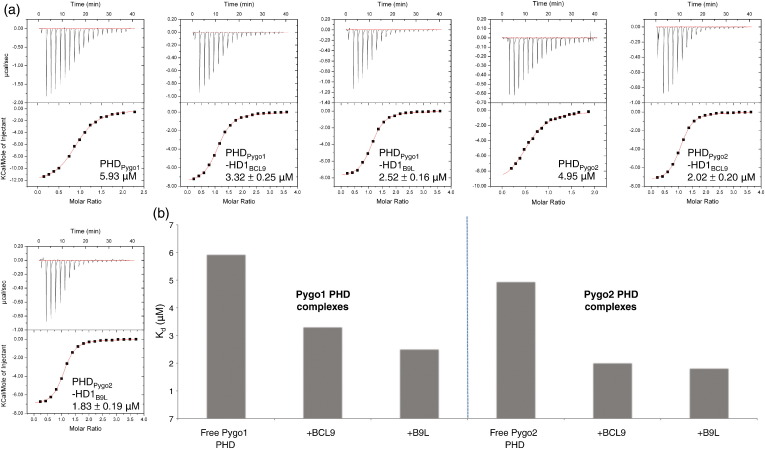
Histone binding affinities of different PHD–HD1 paralog complexes. (a) ITC profiles for H3K4me2 binding of free PHD or PHD–HD1 complexes, as indicated. Data were fitted to a one-site model with the Origin software provided by the manufacturer. The mean *K*_d_ values for each interaction are given in each panel, with standard deviations indicated (*n* = 3 for HD1_BCL9_ complexes; *n* = 4 for HD1_B9L_ complexes); values were calculated from experimental data from independent experiments, thus validating the reproducibility and significance of differences between the paralog complexes. (b) Histogram showing the mean *K*_d_ values.

We generated complexes with each domain combination (see Materials and Methods) and used ITC to compare their binding affinities for a 15-mer H3K4me2 peptide, the preferred histone tail peptide substrate for both human PHD_Pygo1_–HD1_BCL9_ and *Drosophila* PHD_Pygo_–HD1_Lgs_.^13^ The two free PHD fingers exhibit similar affinities for H3K4me2 (*K*_d_ = 5.93 μM and 4.95 μM for PHD_Pygo1_ and PHD_Pygo2_, respectively; Fig. 2a), as expected from their sequence identity with regard to their histone-pocket-lining residues (Fig. 1; see also the text below). However, their affinities for H3K4me2 are increased two to three times by their binding to HD1_BCL9_ and HD1_B9L_ (Fig. 2b), consistent with previous results^13^ (note that the absolute values in Fig. 2 are not absolutely identical with those previously obtained,^13^ likely due to technical differences in peptide quantification and instrumentation in the two different laboratory setups; importantly though, each set of measurements is comparable internally, and both sets show the same ∼ 2× increase from free PHD_Pygo1_ to PHD_Pygo1_–HD1_BCL9_). Interestingly, the two complexes containing PHD_Pygo2_ exhibit a higher affinity for H3K4me2, whereby the highest was measured for PHD_Pygo2_–HD1_B9L_ (*K*_d_ = 1.83 μM; Fig. 2a). Although these differences in affinities are relatively small, they are genuine since they are highly reproducible, with negligible variations between individual measurements (based on different protein preparations; Fig. 2a), and the *n* values determined were consistently close to 1, supporting accurate concentration determination, biologically completely active material and a 1:1 stoichiometry. Thus, the HD1-interacting surface of PHD_Pygo2_ may be more effective than that of PHD_Pygo1_ in communicating the effects of HD1 binding to the histone-interacting surface.

### The structure of the human PHD_Pygo2_–HD1_B9L_ complex

**Fig. 3:**
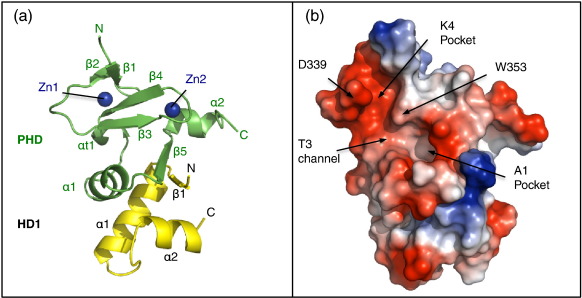
Structure of the human PHD_Pygo2_–HD1_B9L_ complex. (a) Ribbon representation of PHD_Pygo2_–HD1_B9L_ solved at 1.9 Å resolution. (b) Electrostatic surface representation, with orientation as in (a), a K4 pocket lip residue (D339) and the histone-pocket divider (W353) labelled (note that the latter is distinct from the PHD signature residue W377, which is invisible in this view of the histone-binding surface). Key residues and binding sites have been labelled. Electrostatic colouring was calculated with PyMOL.

To further explore the underlying molecular mechanism of this communication, we solved the crystal structure of the human PHD_Pygo2_–HD1_B9L_ complex. The purified complex yielded crystals under multiple conditions, and its structure was solved at 1.9 Å resolution (Fig. 3; Table 1). The overall structure is very similar to that of PHD_Pygo1_–HD1_BCL9_ (with a 0.593 Å rmsd for the core C^α^ backbone  compared to that of 2VPB^13^). Like other PHD fingers, PHD_hPygo2_ binds to two Zn^2+^ in a cross-braced conformation involving two pairs of anti-parallel β-strands, followed by an α-turn and another β-strand flanked by two α-helices (Fig. 3a). It exhibits two distinct protein-interacting surfaces on opposite sides: one for histone binding (Fig. 3b) and another for HD1 binding (see the text below).

The histone-binding surface displays two adjacent deep hydrophobic pockets (for methylated K4 and A1, respectively) divided by a tryptophan (W353), which are linked by the narrow T3 channel (Fig. 3b), as previously described for PHD_Pygo1_–HD1_BCL9_.^13^ In the structure presented here, the K4 pocket is occupied by the presence of a pseudo-ligand (K386, from a symmetry-related protein) that may coordinate the residues surrounding the pocket (Supplementary Fig. 1), reminiscent of a previously observed pseudo-ligand interaction (2VPD^13^), suggesting a propensity of this pocket to be occupied when crystallised. As expected from the sequence identities between the two Pygo paralogs (Fig. 1a), the structural features of their histone binding pockets are very similar.

HD1_B9L_ is a compact domain that consists of an N-terminal β-strand, followed by two α-helices connected by a short loop that allows folding back of α2 towards α1 (Fig. 3a). HD1 interacts with PHD_Pygo2_ in much the same manner as described for PHD_Pygo1_–HD1_BCL9_,^13^ being mediated by two sets of interactions (hydrogen bonds and hydrophobic) of specific residues, all but two of which are identical between the two complexes. Of the hydrogen bonds, four involve main-chain interactions within a β-sheet formed between β-strands from PHD (β5; residues S374, V376 and A378) and HD1 (β1; residues Y236 and F238). A further three hydrogen bonds form between the following PHD and HD1 residues, respectively: W377-T240 (side chain to side chain), G360-N244 (main chain to side chain) and T362-N244 (main chain to side chain) (Supplementary Fig. 2). The hydrophobic interactions involve side chains of specific conserved or semi-conserved residues (Fig. 1b), several of which are critical for binding between Pygo1 and BCL9/Lgs, as previously shown.^37^

The main difference between the structure shown here and that of the PHD_Pygo1_–HD1_BCL9_ complex is the presence of an extra helix (α2) in B9L HD1 (Fig. 3a) that was not properly formed in previous structures, perhaps as a result of crystal contacts. Despite the absence of this helix in earlier structures, the importance was noted, since their deletion abolished PHD–HD1 binding.^13^

**Fig. 4:**
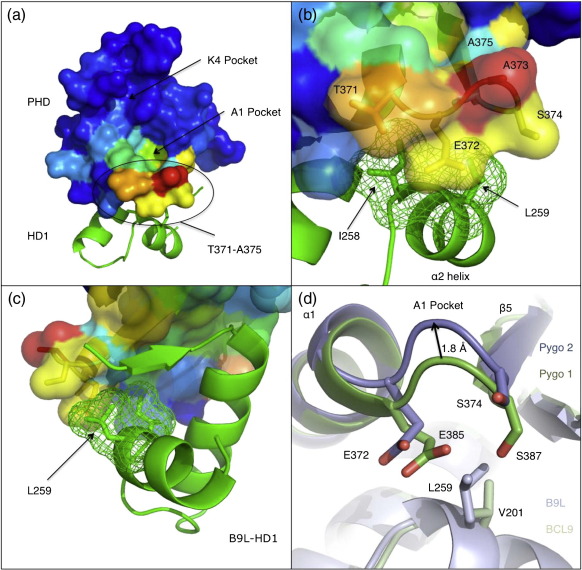
Buttressing of the Pygo2 A1 pocket by the α2 helix of B9L HD1. (a–c) Surface (PHD) and ribbon (HD1) representations of PHD_Pygo2_–HD1_B9L_. Colouring of PHD residues is in accordance with chemical shift perturbations (Fig. 6b), whereby blue indicates no shift perturbation upon HD1 binding and warmer colours show increasing shift perturbations, with red representing the largest. (d) Ribbon and stick representation of the PHD–HD1 interface. Dark and light blue, PHD_Pygo2_ and HD1_B9L_, respectively; dark and light green, PHD_Pygo1_ and HD1_BCL9_, respectively. V201 > L259 causes a 1. 8-Å shift in the main-chain position of A373, which, together with its flanking residues in the loop between α1 and β5, forms the base and sides of the A1 binding pocket. Key residues are labelled.

We note two important features of α2. Firstly, two conserved residues of this helix (I258 and Y261), together with conserved or semi-conserved residues of β1 (F238), α1 (L242, A246, A249 and V250) and the loop linking α1 to α2 (A255), form the hydrophobic core of HD1, which likely stabilises its interaction with the PHD. Secondly, α2 is positioned so that I258 and L259 are in contact with the base of the A1 pocket, namely, with PHD residues T371-A375, which flank the connecting loop between α1 and β5 (Fig. 4a–c), apparently buttressing the pocket.^13^ α2 is thus an essential structural feature of HD1_B9L_, directly supporting the A1 pocket base of PHD_Pygo2_.

Two amino acid substitutions between the two HD1 paralogs could be functionally significant for their interaction with PHD. Firstly, V201 of HD1_BCL9_ is substituted by L259 in HD1_B9L_ (Fig. 1b); the latter forms direct contacts with the A1 pocket base (see the text above) by forming close hydrophobic interactions with Pygo2 E372, S374 and A375 (Fig. 4b). However, to accommodate the smaller V201 in PHD_Pygo1_–HD1_BCL9_ (without significant loss of hydrophobic contacts), the PHD loop that forms the A1 pocket base appears to shift by ∼ 1.8 Å, judging by the superimposed structures of PHD_Pygo1_–HD1_BCL9_ and PHD_Pygo2_–HD1_B9L_ (Fig. 4d). This change could contribute to the different histone binding affinities seen in different PHD–HD1 complexes.

**Fig. 5:**
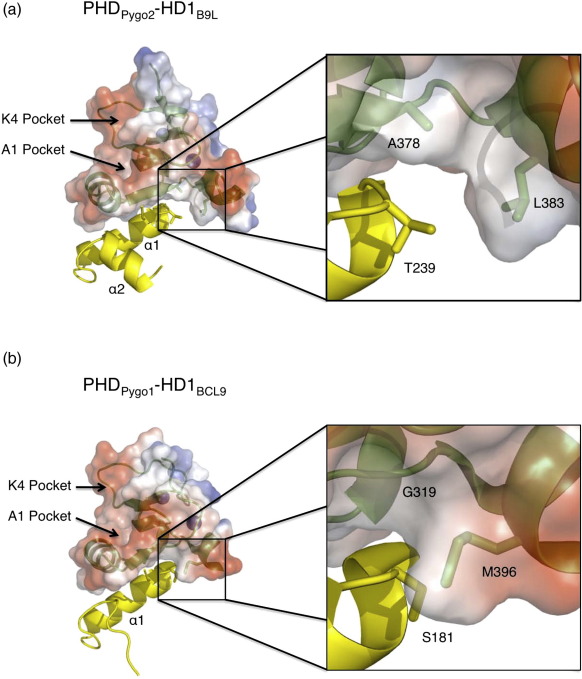
Paralog-specific PHD–HD1 interfaces. Surface (PHD) and ribbon (PHD and HD1) representations of (a) PHD_Pygo2_–HD1_B9L_ and (b) PHD_Pygo1_–HD1_BCL9_; the former are shown as transparent electrostatic surface potential representations (calculated with PyMOL). Green, PHD; yellow, HD1. Close-up images illustrate the PHD ‘clamps’ at the PHD–HD1 interface.

Secondly, S181 of HD1_BCL9_ is substituted by T239 in HD1_B9L_, which faces a PHD surface that also exhibits two substitutions: G391 > A378 and M396 > L383 (Pygo1 > Pygo2) (Fig. 1). The variant residues in this interface cause significant changes in the hydrophobic interactions seen in the two different complexes (Fig. 5), which are likely to affect the affinities between the different PHD and HD1 paralog domains (which we were unable to determine due to technical difficulties with purifying HD1 whose surface is highly hydrophobic). Importantly, none of these interactions directly involves any of the histone-binding residues; however, they may influence histone binding indirectly (see the text below) by affecting the affinity between PHD and HD1.

### Binding of HD1 to PHD triggers allosteric remodelling of the T3 channel

**Fig. 6:**
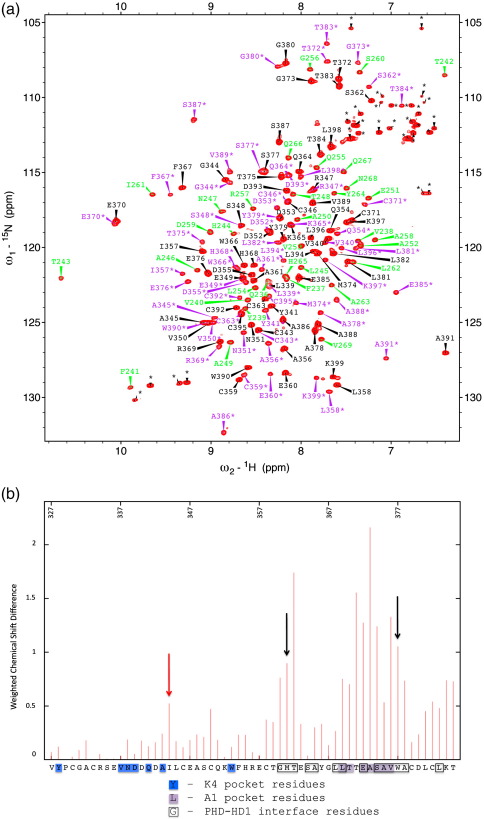
Chemical shift perturbations of PHD residues triggered by HD1 binding. Assigned HSQC spectrum for free PHD_Pygo2_ (black) and PHD_Pygo2_–HD1_B9L_ complex (purple). Green, HD1_B9L_ (in complex). (b) Chemical shift difference map showing backbone N–H chemical shift differences between free PHD_Pygo2_ and the PHD_Pygo2_–HD1_B9L_ complex, as calculated from the HSQC spectrum shown in (a); weighted chemical shift differences represent absolute values of [change in ^1^H shift] + [change in ^15^N shift/5].^38^ Underneath the graph is the primary sequence of Pygo2, with boxings and colourings as indicated. Red arrow, I344 (allosteric residue lining the T3 channel); black arrows, M361 and W377 (allosteric residues interacting with HD1 and I344).

Our abovedescribed observations indicate communication between HD1 and histone binding through the PHD finger. To investigate this further, we used NMR spectroscopy as a highly sensitive probe of intermolecular interactions in solution. An ^1^H–^15^N heteronuclear single-quantum correlation (HSQC) spectrum of PHD_Pygo2_, alone or in complex with HD1_B9L_ (Fig. 6a), allowed us to map chemical shift perturbations for specific Pygo2 resonances that are induced by HD1_B9L_ binding to PHD_Pygo2_ in solution (Fig. 6b). These were mapped onto the crystal structure (Fig. 4a–c). As expected, the largest set of shift perturbations originates from PHD residues that are directly in contact with HD1 in the crystal structure (Fig. 6b, boxed residues). These include residues that line the A1 pocket (Fig. 6b, highlighted in purple; Fig. 4a), implying that this pocket may undergo some remodelling upon HD1 binding to PHD. In addition, we observe several perturbations of PHD residues that do not interact directly with HD1 in the crystal structure, but are linked immediately to an HD1-interacting residue (e.g., A373). By contrast, there are no significant shift perturbations of PHD residues that form the K4 pocket, despite the fact that the K4 pocket lip residue D339 (of the EVND motif in the ‘Pygo loop’^34^) is highly flexible in the crystal structure (Fig. 6b, highlighted in blue; Fig. 4a). Thus, the structure of the K4 pocket is not affected by HD1 binding to PHD.

The most interesting shift perturbations are those of PHD residues that are not directly in contact with HD1, but contribute to a histone binding pocket. One such residue is I344, which lines the T3 channel: I344 shows a highly significant chemical shift perturbation (Fig. 6b, red arrow; ∼ 13× above the significance threshold of 0.04 ppm^38^), indicating a change in the chemical environment of its main-chain N–H group. We conclude that the environment of I344 must be affected *indirectly* by HD1 binding, implying that I344 participates in an allosteric interaction. The only other examples of this class of ‘relayed’ shift perturbations that we observed are those of two zinc-coordinating cysteine residues (C350, C4 coordinating Zn2; C358, C5 coordinating Zn1; Fig. 3a), which may represent movements in the structural PHD core accommodating the allosteric communication through this core (see the text below).

**Fig. 7:**
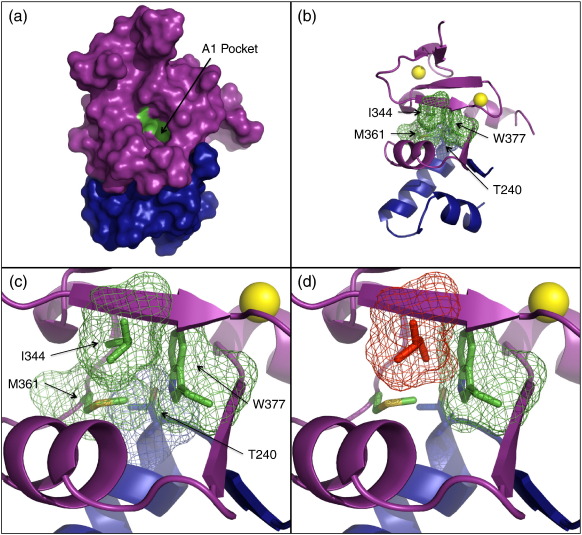
Allosteric residues within the PHD_Pygo2_ core. (a and b) Identical views of PHD_Pygo2_–HD1_B9L_ (purple, PHD; blue, HD1) in (a) surface and (b) ribbon representations, with locations of PHD I344, M361 and W377 (green), and invariant HD1 T240 (blue) indicated. Yellow, Zn^2+^. (c) Ribbon representation of close-up view, with mesh surface representations of the side chains of the allosteric PHD residues (green) interacting with T240 (blue). I344 is shown as an ‘unfavoured’ rotamer, calculated from the experimentally determined electron density (Supplementary Fig. 4). (d) Same view as (c), with I344 (red) in the ‘normal’ rotamer configuration, which would produce a steric clash with W377.

How does I344 communicate with the HD1 binding interface of the PHD finger? The crystal structure shows that I344 is within the PHD_Pygo2_ core, lying against the side chains of two residues (M361 and W377) that interact directly with HD1 (Fig. 7a–c): M361 forms hydrophobic interactions with the side chain of HD1 T240, which also forms a hydrogen bond with the side chain of W377. T240 therefore seems critical to both the PHD–HD1 interaction and the allosteric communication. Interestingly, this residue is not only conserved between the two human paralogs (Fig. 1) but invariant among all known BCL9 orthologs in other species. As expected, PHD M361 and W377 themselves also show significant chemical shift perturbations upon HD1 binding (Fig. 6b, black arrows), providing further evidence for an interaction chain running through the PHD core, which could provide a link between HD1 and histone binding. Notably, W377 corresponds to the PHD signature residue, defining this particular class of Zn-liganded domains (see Introduction).

A further interesting point concerns the chemical shift perturbations of the abovementioned PHD residues T371-S374. Of these, only E372 and S374 directly interact with HD1, through hydrophobic interactions with I258 and L259. Despite not directly interacting with HD1, T371 and A373 show very large chemical shift perturbations upon HD1 binding (maximal for A373), which can thus be described as ‘passive’ perturbations. A373 is positioned within a short loop between PHD α1 and β5, two structural elements each of which engages in direct contacts with HD1. Our NMR data suggest that the structure of this short loop is responsive to HD1 binding: indeed, it appears that HD1 binding may reorientate the backbone carbonyl groups of PHD L369, E372 and A375 to shape the deep A1 pocket such that it is capable of hydrogen bonding with the incoming N-terminal amino group of the histone tail peptide. This provides experimental support for our previous proposal that HD1 supports PHD to shape the A1 pocket base.^13^

### The residues participating in allosteric communication through the PHD core are critical for histone H3 tail binding

To test whether I344 and W377 contribute to the affinity of Pygo2 PHD for histone H3 tails, we designed mutations in these residues. This was not straightforward, since both their side chains contribute to the structural PHD core. We thus used the crystal structure to model minimal alterations compatible with preserving the structural integrity of the core. I344 was mutated to alanine to remove its physical interaction with the side chain of W377 while retaining the hydrophobic nature of its side chain and its interaction with H3T3. W377 was mutated to a phenylalanine, a known naturally occurring variant of this PHD signature residue in a small subset of PHD fingers^34^—a substitution that is expected to maintain the structural integrity of the PHD core^35,36^ while removing the physical interaction between W377 and I344. Since the cores of the two PHD paralog domains are structurally very similar to each other, we introduced the two mutations into both PHD_Pygo2_–HD1_B9L_ (W377F and I344A) and PHD_Pygo1_–HD1_BCL9_ (W390F and I357A) to determine their affinities for H3K4me2 by ITC.

**Fig. 8:**
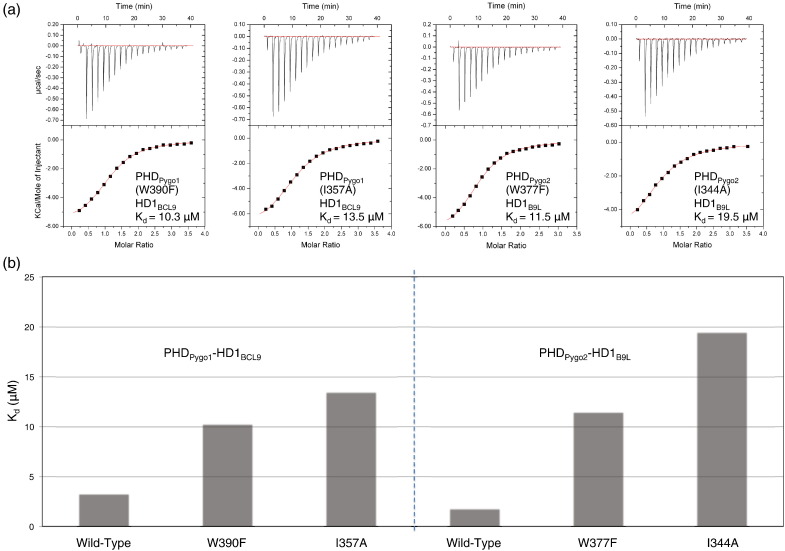
Histone binding affinities of mutant PHD–HD1 complexes. ITC profiles and histograms as in Fig. 1 (including data fitting and *K*_d_ values) of mutant PHD–HD1 complexes as indicated in (a) and (b).

Each mutation causes a marked decrease in H3K4me2 affinity, with W390F and W377F reducing the affinity to 10.3 μM and 11.5 μM, respectively, and with I357A and I344A reducing the affinity to 13.5 μM and 19.5 μM, respectively (Fig. 8). These values are somewhat lower than those measured for the corresponding free PHD fingers (Fig. 2) (i.e., without allosteric remodelling), suggesting that our designer point mutations may also affect the structural PHD cores, which could contribute to the observed reductions in PHD–H3K4me2 binding affinities. Regardless, the results from these mutant complexes clearly indicate the functional importance of I357/344 and W390/377 in histone tail binding.

To examine the structural integrity of the mutant complexes, we subjected the PHD_Pygo1_–HD1_BCL9_ complexes to analysis by circular dichroism (CD). Reassuringly, the CD spectra indicate that the secondary structure content of both mutant complexes was the same (judging by the constant helical CD signals at 208 nm and 222 nm) as that observed for the wild-type (wt) complex at 22 °C (Supplementary Fig. 3a; note that the change in CD spectra between W390F and the other two proteins, centered around 230 nm, can be explained in terms of an aromatic contribution to the far-UV spectrum that is lost on tryptophan mutation). We also carried out a CD thermal denaturation analysis of both mutant complexes, which revealed somewhat lower *T*_m_ values for the two mutant complexes compared to wt, indicating reduced thermal stabilities of the mutants at high temperature (Supplementary Fig. 3b); this is not unexpected, given that the mutations are in the PHD core. Importantly though, there are no detectable differences between wt and mutant complexes throughout the range of physiological temperatures (below 40 °C). Finally, one-dimensional ^1^H NMR analysis revealed that the spectra of wt and mutant complexes are essentially the same (Supplementary Fig. 3c), providing additional evidence that the structural fold of the PHD–HD1 complex is not detectably perturbed by the W390F and I357A mutations, and that the wt and mutant complexes are equally folded at the temperature of our ITC measurement (25 °C). Taken together, these data argue against the notion that the reduced affinities of the mutant complexes for histone H3 tail peptides solely reflect structural deformations or compromised stabilities.

## Discussion

We have shown that, of the four possible PHD–HD1 paralog complexes, PHD_Pygo2_–HD1_B9L_ has the highest histone binding affinity, while PHD_Pygo1_–HD1_BCL9_ has the lowest histone binding affinity. B9L may thus be a more potent Pygo co-factor than BCL9, and Pygo2 may be more responsive to its co-factors than Pygo1—the caveat being that the measured differences in affinities are small. Notwithstanding this, we propose that the ∼ 2× difference in histone binding affinity between PHD_Pygo2_–HD1_B9L_ and PHD_Pygo1_–HD1_BCL9_ (Fig. 2) may be determined by two sets of interactions at the PHD–HD1 interface that involve paralog-specific residues, which could impact on the histone binding pockets directly or indirectly through allosteric remodelling (see the text below). Importantly, a 2-fold difference in binding affinity could be physiologically significant *in vivo* if the biologically relevant protein concentrations were within the range of the measured *K*_d_. While there is currently no available information that would allow us to estimate these concentrations, we note that Pygo2 is readily detectable in murine epithelial cells,^25^ like other Wnt signalling components with cellular concentrations in the submicromolar range.^39^ A striking precedence for the physiological relevance of a 2-fold effect is found in sex determination and dosage compensation.^40^

One set of paralog-specific interactions relates to the substitution of V201 (BCL9) for L259 (B9L), which brings about a small shift of the main-chain position of a critical residue (A373 in Pygo2) contributing to the A1 pocket base. This V > L change therefore impacts directly on the shape of this pocket (Fig. 4d). The L259 residue of B9L thus appears to buttress the A1 pocket more effectively than its BCL9 V201 counterpart, consistent with the notion that B9L may be a more potent Pygo co-factor than BCL9.

**Fig. 9:**
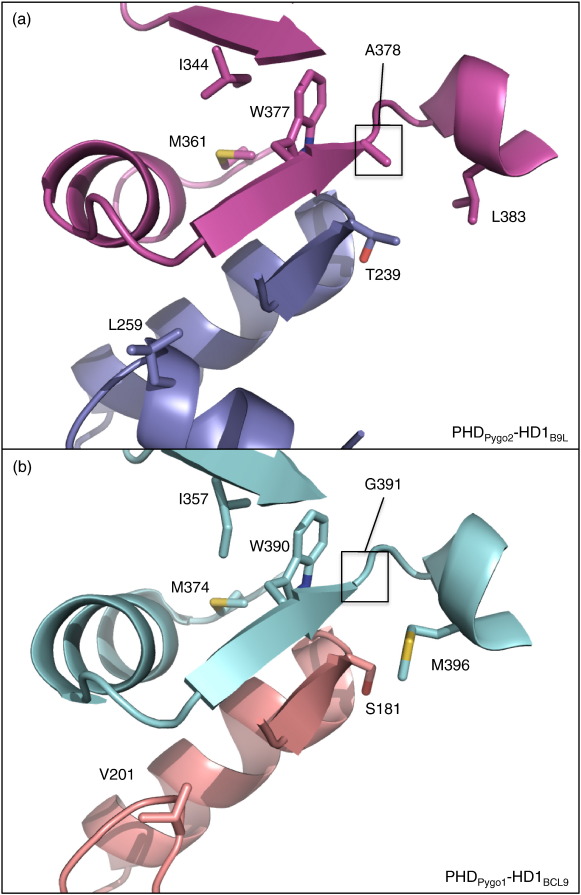
Paralog-specific residues in the PHD clamp region that abuts the PHD tryptophan signature residue. Ribbon and stick (key side chains) representations of (a) PHD_Pygo2_–HD1_B9L_ and (b) PHD_Pygo1_–HD1_BCL9_, focusing on the PHD–HD1 interface in relation to PHD allosteric residues. Magenta, PHD_Pygo2_; blue, HD1_B9L_; cyan, PHD_Pygo1_; pink, HD1_BCL9_.

The second set of paralog-specific residues at the PHD–HD1 interface (G391/A378, M396/L383 and S181/T239) clusters in the same PHD region, forming a ‘clamp’ at one end of its otherwise flat HD1 binding interface (Fig. 5). They interact with one another via hydrophobic interactions, which are likely to impact on the local architecture of this region, and may also affect the PHD–HD1 binding affinities. For example, Pygo2 L383 is expected to interact more effectively with B9L T239 compared to BCL9 S181, clamping the interaction with B9L. Importantly, A378 and G391 are directly linked to the PHD signature residue (W377/W390) that contributes to the structural PHD core (Fig. 9) and thus could provide a critical input to the allosteric communication relayed by this PHD signature residue (see the text below).

Our NMR data provide experimental ‘in-solution’ evidence that the two highly conserved residues W377 and I344 interact to relay an allosteric communication through the structural core of the PHD finger triggered by HD1 binding. The X-ray structure of PHD_Pygo2_–HD1_B9L_ provides an explanation for this short allosteric reaction chain (Fig. 7b and c): W377/W390 is directly linked to Pygo2 A378 (or Pygo1 G391) in the abovementioned clamp region. Variations in the architecture of this region, brought about by the different combinations of paralog-specific PHD and HD1 residues, may thus impact slightly differently upon this tryptophan (Fig. 9); in the case of B9L, this could enhance the allosteric communication, which may also be assisted by the hydrogen bonding between W377 and HD1 T240. The latter may be boosted further by the B9L paralog-specific T239, possibly a more potent allosteric inducer than its BCL9 equivalent S181, since T239 mediates strong hydrophobic interactions with other residues in this area.

A second possible input for the allosteric communication is provided by a common component of both Pygo PHD fingers—the conserved M361 (M374 in Pygo1), which forms part of the hydrophobic PHD–HD1 interface and directly contacts I344 in the structural PHD core (Fig. 7b and c). Our NMR data reveal a large chemical shift perturbation of M361 upon HD1 binding, consistent with M361 modulating the shape of the T3 channel indirectly through this interaction with I344.

Our notion of an allosteric communication is further supported by an intriguing clue derived from our crystal structure, in which I344 is flagged as being an incorrect rotamer in both asymmetric units. However, investigation of this residue and its surrounding electron density established unambiguously that the conformation of this side chain is correctly calculated from the experimental data (Supplementary Fig. 4). It appears to result from a constraint exerted by the side chain of W377 (Fig. 7c), which would clash sterically with the normal rotamer configuration of I344 (Fig. 7d). Its ‘unnatural’ configuration thus appears to be imposed by W377, and its close contact with I344 may allow this critical residue to be particularly responsive to any shift of W377 upon HD1 binding.

Based on our data, we propose that the conserved I344 acts as the single ‘output’ residue of an allosteric communication initiated by two conserved ‘input’ amino acids, M361 and the PHD tryptophan signature residue; in turn, both input residues form direct interactions with T240, an invariant threonine residue present in all BCL9 orthologs (Fig. 7b). We envisage that HD1 binding to PHD triggers small local changes in the side-chain conformations of M361 and W377, as a result of direct interactions with HD1 T240, which enable them to impact on I344 in the PHD core, triggering changes in the conformation of the isoleucine side chain and thereby remodelling the surface of the T3 channel. This ‘two-input one-output’ model for allostery in the PHD finger may also help to explain why the I > A mutations caused larger reductions in affinities than the W > F mutations (Fig. 8): the latter are expected to retain a partial allosteric input by M361, while the former disable the single allosteric ‘output’ residue, thus abolishing the entire allosteric communication.

In summary, our NMR data provided evidence that the Pygo2 PHD finger is an allosterically modulated domain capable of transducing a signal from one of its ligand surfaces to the other—from its HD1 to its histone binding site. This allosteric communication is transmitted through the structural PHD core and involves interaction between three conserved residues (I344, M361 and W377) and so is likely to also occur in Pygo1; it targets the T3 channel, which appears to undergo subtle reshaping as a result. Further analysis is required to determine the precise mechanism of this allosteric communication and its impact on histone binding (e.g., we do not know whether the allosteric trigger clicks the T3 channel into an alternative stable conformation, or whether it causes short-lived reshaping and/or stabilises its conformation). Whatever the case, the allosteric communication is complemented by a direct interaction of HD1 with PHD residues that constitute the A1 pocket base, possibly modulating the shape and/or stability of this pocket that anchors the N-terminus of the histone H3 tail. The net outcome of these two effects is an enhancement of the affinity of Pygo's PHD finger for methylated histone H3. Notably, although we have not been able to determine this experimentally, we would expect this communication to be bidirectional on thermodynamic grounds; in other words, the binding of PHD to histone H3 tail could also increase Pygo's affinity for BCL9, ultimately boosting its capture of β-catenin, as previously suggested.^12^

The role of the PHD signature residue in mediating an allosteric interaction is intriguing. To our knowledge, Pygo PHD is the only known PHD finger that is capable of interacting simultaneously with two functional ligands.^13^ However, its structural core is similar to that of other PHD fingers,^35,36^ as is the layout of its histone binding pockets.^15–17^ Other PHD fingers also possess a putative second interaction surface equivalent to Pygo's HD1-interacting surface (‘loop 2’ surface^34^). It is thus conceivable that other PHD fingers also depend on specific co-factors for their histone-decoding activities.

## Materials and Methods

### Expression and purification of proteins

For the crystal structure, PHD_Pygo2_–HD1_B9L_ was generated from a construct spanning MBP-tagged PHD [amino acids 325–387, separated from MBP via a tobacco etch virus (TEV) protease site] linked via seven amino acids (a linker of alternating glycines and serines) to HD1 (amino acids 233–266). The complex was expressed in *Escherichia coli* B834(DE3) cells and purified by Ni-NTA resin, TEV cleavage, HiTrap Q anion-exchange chromatography and size-exclusion chromatography. For ITC, 6×His-tagged PHD_Pygo1_–HD1_BCL9_ and PHD_Pygo2_–HD1_B9L_ complexes (containing Pygo1 residues 340–400 or Pygo2 residues 327–387, separated from the His tag via a TEV protease site and linked, as described, to BCL9 residues 177–205 or B9L residues 235–263) were expressed in *E. coli* BL21(DE3) RIL cells and purified by Ni-NTA and size-exclusion chromatography. The purity of all proteins was assessed by SDS-PAGE prior to use.

### Crystallisation

Crystallisation conditions were optimised with LMB crystallisation condition screens in 96-well sitting-drop format using 100-nl drops.^41^ Crystals were grown at 19 °C by the vapour diffusion method and emerged after ∼ 24 h under multiple conditions. The crystals used for data collection were taken from the following conditions: 6% polyethylene glycol 1000, 1.6 M (NH_4_)_2_SO_4_, 100 mM Hepes (pH 7.5) and 45 mM HCl. Crystals were soaked in a cryobuffer containing the aforementioned chemicals plus 25% glycerol for < 1 min before flashcooling in liquid nitrogen.

### Diffraction data collection and structure solution

Diffraction data were collected at the European Synchrotron Radiation Facility. Structures were solved by molecular replacement with Phaser^42^ based on the PHD_Pygo1_–HD1_BCL9_ complex (2VP7^13^; Table 1). Crystallographic data were processed with Mosflm^43^ and scaled with Scala.^44^ The structures were refined with Refmac,^45^ and the models were updated with Coot.^46^ Analysis of the structures was performed with CCP4i programmes,^47^ and images were created with PyMOL. The structures were validated with MolProbity.^48^

### ITC measurements

ITC was carried out (at 25 °C) with an iTC 200 Microcalorimeter (GE Healthcare) following dialysis of purified wt and mutant His-tagged PHD_Pygo1_–HD1_BCL9_ and PHD_Pygo2_–HD1_B9L_ complexes in 25 mM Tris (pH 8.0) and 100 mM NaCl. Titrations consisted of 19 consecutive  2-μl injections of peptide solution (following a pre-injection of 0.5 μl) into the protein at time intervals of 120 s or 150 s. The 15-mer H3K4me2 histone tail peptide was used as previously described;^13^ its concentration was determined by amino acid analysis.

### NMR spectroscopy

For NMR studies, PHD_Pygo2_ and HD1_B9L_ were co-expressed in minimal medium supplemented with [^15^N]ammonium chloride and [^13^C]glucose as the sole nitrogen and carbon sources, and a complex was purified as previously described.^13^ All NMR spectra were recorded with a Bruker DRX spectrometer operating at 600 MHz ^1^H frequency, equipped with a triple-resonance inverse cryogenic probe head at a sample temperature of 25 °C. The sample contained both free (∼ 380 μM) and HD1-bound (∼ 110 μM) PHD at pH 6.7 in 50 mM aqueous Tris buffer. Backbone resonance assignments were obtained with standard triple-resonance techniques (HNCACB, CBCA(CO)NH, HNCO and HN(CA)CO). For chemical shift mapping, a fast HSQC spectrum^49^ was obtained with 1024 and 192 data points in *t*_2_ and *t*_1_, respectively, with spectral widths of 8333 Hz and 1825 Hz. The number of points in *t*_1_ was doubled by forward complex linear prediction prior to Fourier transformation. Spectra were processed with TopSpin version 2 (Bruker) and analysed using Sparky version 3.110 (Goddard T. D. & Kneller D. G., SPARKY 3, University of California, San Francisco).

### Far-UV CD spectroscopy

Far-UV CD spectra and thermal melts of wt and mutant His-tagged PHD_Pygo1_–HD1_BCL9_ (26 μM) were recorded with a Jasco J815 CD spectrophotometer thermostated with a Peltier-controlled cell block (Jasco). Eight accumulations were recorded and corrected for the buffer signal. All protein samples were dialysed into a buffer of 25 mM phosphate (pH 6.7) and 50 mM NaCl prior to CD spectroscopy.

### Accession code

Coordinates and structure factors have been deposited with the Protein Data Bank under accession code 2XB1.

## Figures and Tables

**Table 1:** Data collection and refinement statistics

*hPHD2_B9L_HD1*	
Beamline	ID29 (European Synchrotron Radiation Facility)
Strategy (°)	90
Wavelength (Å)	1.04
Space group	*P*2_1_2_1_2_1_
Unit cell parameters	
*a*, *b*, *c* (Å)	38.75, 69.73, 82.07
α, β, γ (°)	90, 90, 90
Resolution (Å)	38.75–1.900 (2.00–1.90)^a^
*R*_merge_ (%)^b^	8.4 (36.0)
*I*/σ(*I*)	9.4 (2.9)
Completeness (%)	99.5 (99.5)
Multiplicity	3.4 (3.4)
Complexes (a.u.)	2

*Refinement*	
Resolution (Å)	31.31–1.90 (1.949–1.900)
Number of reflections	16,788
Test set size (%)	7.1
*R*_work_ (%)	17.122 (22.9)
*R*_free_ (%)	21.715 (26.0)
Number of atoms (non-H)	1721
Residues (PHD/HD1)	325–389/235–266
〈*B*〉 (Å^2^)	9.815
rmsd	
Bond length (Å)	0.021
Bond angle (°)	1.599
Ramachandran plot	
In favoured regions (%)	91.9
In allowed regions (%)	6.9
Outliers (%)	1.2 (S349^c^)

a Values for the highest-resolution shell (Å) are shown in parentheses.

b *R*_merge_ = ∑_*hkl*_|*I*_*hkl*_ − 〈*I*_*hkl*_〉|/∑*_hkl_I_hkl_*.

c S349 is present in an unusual conformation (the electron density is clear) due to the adjacent C350, which forms part of the second Zn^2^^+^ binding site.

## Appendix A

Supplementary Fig. 1 Pseudoligand interaction in the PHD_Pygo2_–HD1_B9L_ complex. Surface representation of PHD–HD1 showing a pseudo-ligand, K386 (orange stick representation), which occupies the K4 binding pocket of the PHD finger. K384 is also highlighted as this residue sits above the A1 pocket. This pseudoligand consists of the C-terminal end of another PHD molecule found in a neighbouring asymmetric unit.

Supplementary Fig. 2 Hydrogen bonds in the PHD–HD1 interface. Ribbon and stick representation of the PHD–HD1 interface. Green, PHD_Pygo2_; blue, HD1_B9L_. All hydrogen bonds are indicated with dotted lines, and bonded residues are labelled.

Supplementary Fig. 3 Structural integrities and thermal stabilities of mutant PHD_Pygo1_–HD1_BCL9_ complexes. (a) Overlaid far-UV CD spectra of wt and mutant PHD_Pygo1_–HD1_BCL9_ complexes recorded at 22 °C. Blue, wt; red, W390F; green, I357A (note that W390 contributes a spectral feature at 230 nm typical of some aromatic residues in a chiral environment, which is thus absent in the W390F mutant). The spectra indicate that the mutations do not perturb the secondary structure compositions of the mutant PHD_Pygo1_–HD1_BCL9_ complexes. (b) Overlaid thermal denaturation UV CD spectra (220 nm) of wt and mutant PHD_Pygo1_–HD1_BCL9_ complexes. A decrease in the thermal stability of the mutant PHD_Pygo1_–HD1_BCL9_ complexes is observable at high temperatures; however, there is no significant difference in their thermal stability at the temperature (25 °C) at which the ITC measurements were obtained. (c) Proton NMR spectra (600 MHz) with W5 WATERGATE solvent suppression of 50 μM protein in phosphate buffer (pH 6.8) at 25 °C. Top, wt; middle, I344A; bottom, W377F.

Supplementary Fig. 4 Calculated electron density for I344 in support of the modelling of I344 as an ‘incorrect’ rotamer. Stick representation of PHD_Pygo2_ centred on the triad of I344, M361 and W377 within the PHD structural core; the image shows the calculated 2*F*_o _− *F*_c_ map contoured at 1.0σ. The electron density unambiguously identifies the conformation of the I344 side chain, despite this being flagged up as an ‘incorrect’ rotamer by the structure validation software MolProbity.
