# Supplementary material for: Allosteric Remodelling of the Histone H3 Binding Pocket in the Pygo2 PHD Finger Triggered by Its Binding to the B9L/BCL9 Co-Factor
Source: J Mol Biol. 2010 Sep 3;401(5-3):969–84. doi: 10.1016/j.jmb.2010.07.007 (PMC2927781; doi:10.1016/j.jmb.2010.07.007)
Supplement: Supplementary Fig. 1 — Pseudoligand interaction in the PHDPygo2–HD1B9L complex. Surface representation of PHD–HD1 showing a pseudo-ligand, K386 (orange stick representation), which occupies the K4 binding pocket of the PHD finger. K384 is also highlighted as this residue sits above the A1 pocket. This pseudoligand consists of the C-terminal end of another PHD molecule found in a neighbouring asymmetric unit. [file mmc1.pdf]

SUPPLEMENTARY FIGURES

Suppl. Fig. 1

Pseudoligand interaction in the PHD_{Pygo2}-$HD1_{B9L}$ complex

Surface representation of PHD-HD1, showing a pseudo-ligand, K386 (orange stick representation), which occupies the K4 binding pocket of the PHD finger. K384 is also highlighted as this residue sits above the A1 pocket. This pseudoligand consists of the C-terminal end of another PHD molecule found in a neighbouring asymmetric unit.

Suppl. Fig. 2

Hydrogen bonds in the PHD-HD1 interface

Ribbon and stick representation of the PHD-HD1 interface; green, PHD_{Pygo2}; blue, $HD1_{B9L}$. All hydrogen bonds are indicated with dotted lines, and the bonded residues are labelled.

Suppl. Fig. 3

Structural integrities and thermal stabilities of mutant PHD_{Pygo1}-$HD1_{BCL9}$ complexes

(**a**) Overlaid far UV-CD spectra of wt and mutant PHD_{Pygo1}-$HD1_{BCL9}$ complexes, recorded at 22°C; blue, wt; red, W390F; green, I357A (note that W390 contributes a spectral feature at 230 nm, typical of some aromatic residues in a chiral environment, which is thus absent in the W390F mutant). The spectra indicate that the mutations do not perturb the secondary structure compositions of the mutant PHD_{Pygo1}-$HD1_{BCL9}$ complexes. (**b**) Overlaid thermal denaturation UV-CD spectra (220 nm) of wt and mutant PHD_{Pygo1}-$HD1_{BCL9}$ complexes; a decrease in the thermal stability of the mutant PHD_{Pygo1}-

$HD1_{BCL9}$ complexes is observable at high temperatures, however there is no significant difference in their thermal stability at the temperature (25°C) at which the ITC measurements were obtained. (**c**) 600 MHz Proton NMR spectra with W5 WATERGATE solvent suppression of 50 µM protein in phosphate buffer pH 6.8, 25°C; top, wt; middle, I344A; bottom, W377F.

Suppl. Fig. 4

Calculated electron density for I344 in support of the modelling of I344 as an 'incorrect' rotamer

Stick representation of PHD_{Pygo2} centred on the triad of I344, M361 and W377 within the PHD structural core; the image shows the calculated $2F_O$-F_C map contoured at 1.0 sigma. The electron density unambiguously identifies the conformation of the I344 side-chain, despite this being flagged up as an 'incorrect' rotamer by the structure validation software MolProbity.

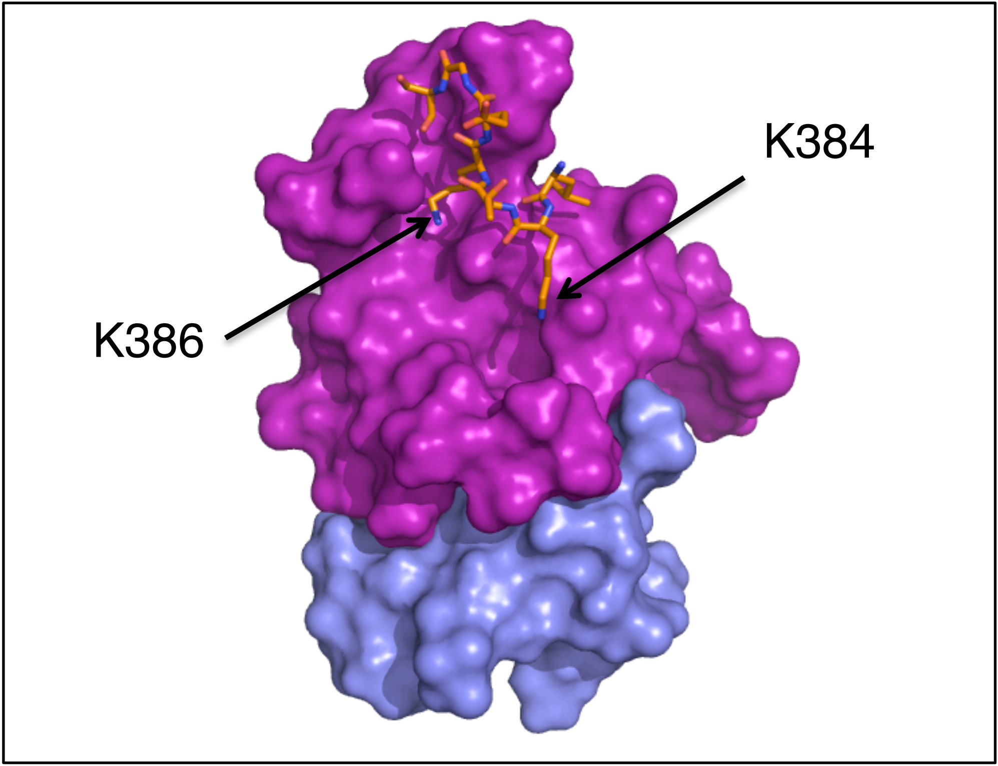

Supplementary Figure 1 Miller et al.

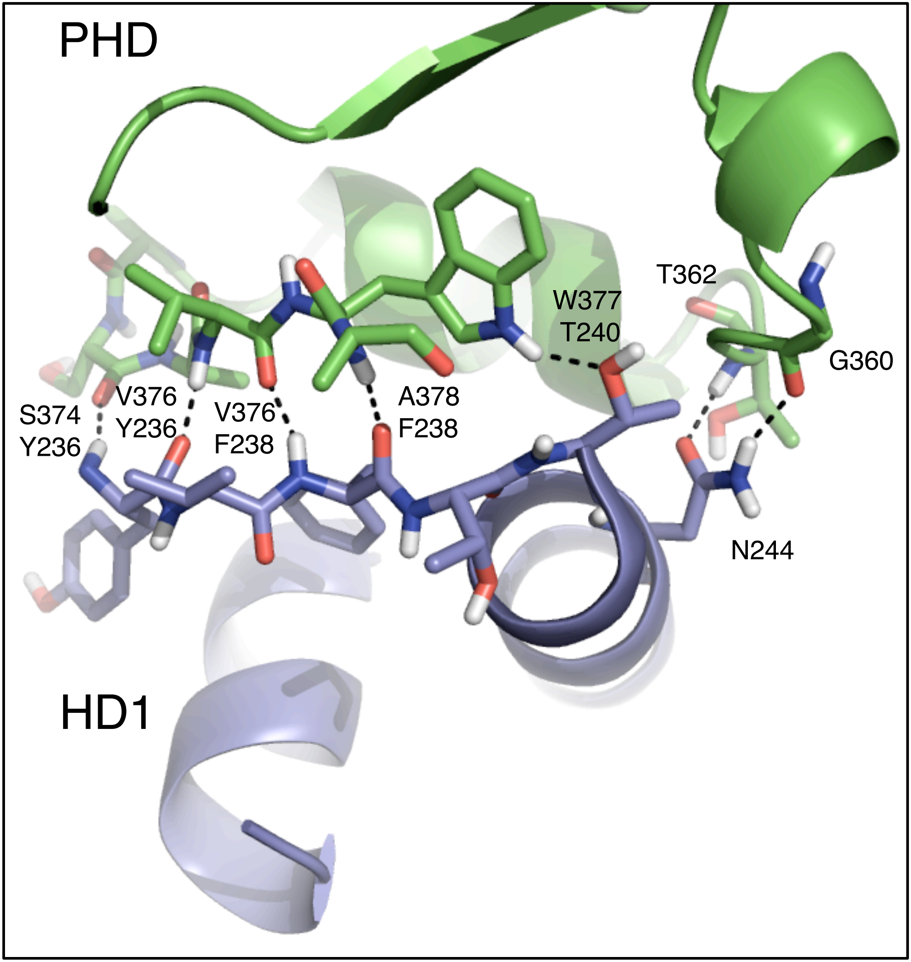

Supplementary Figure 2 Miller et al.

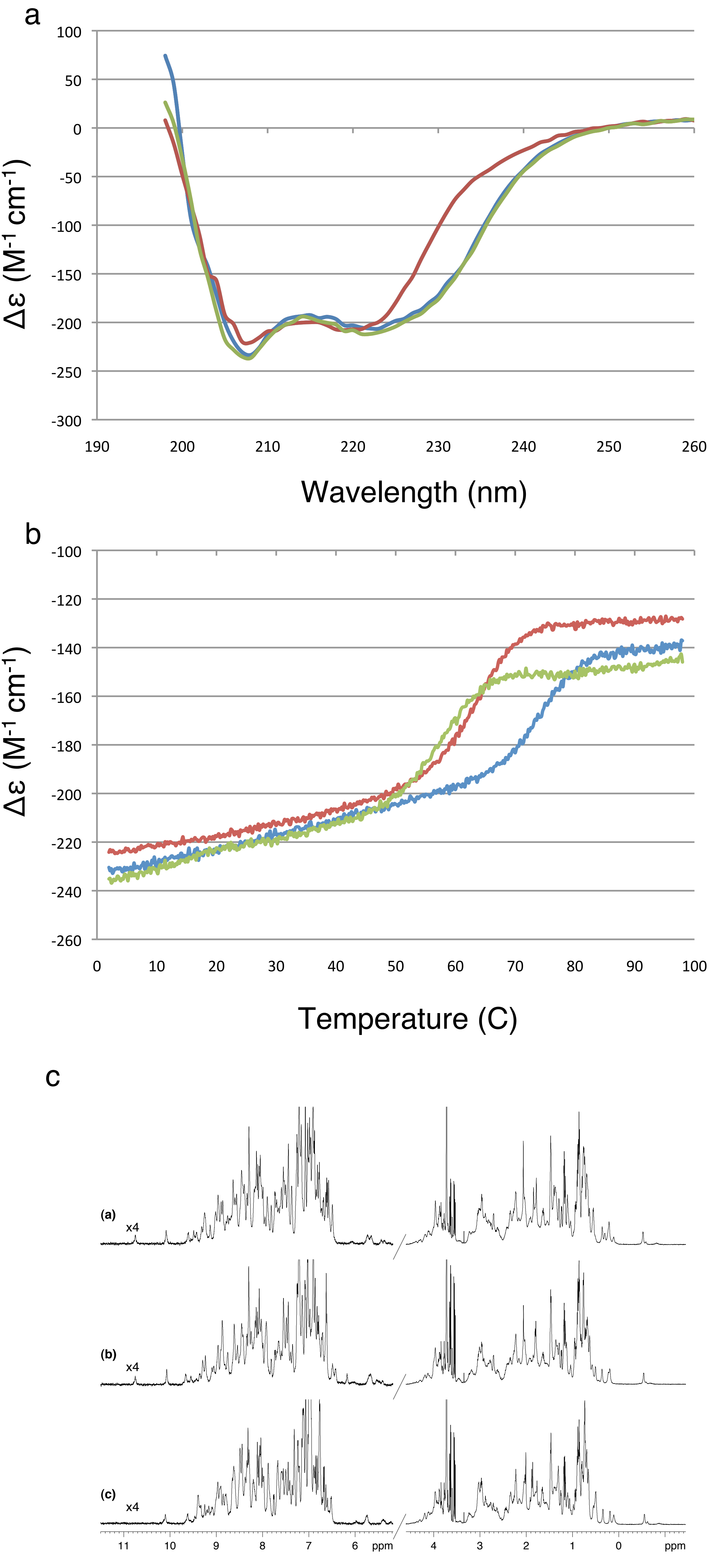

Supplementary Figure 3 Miller et al.

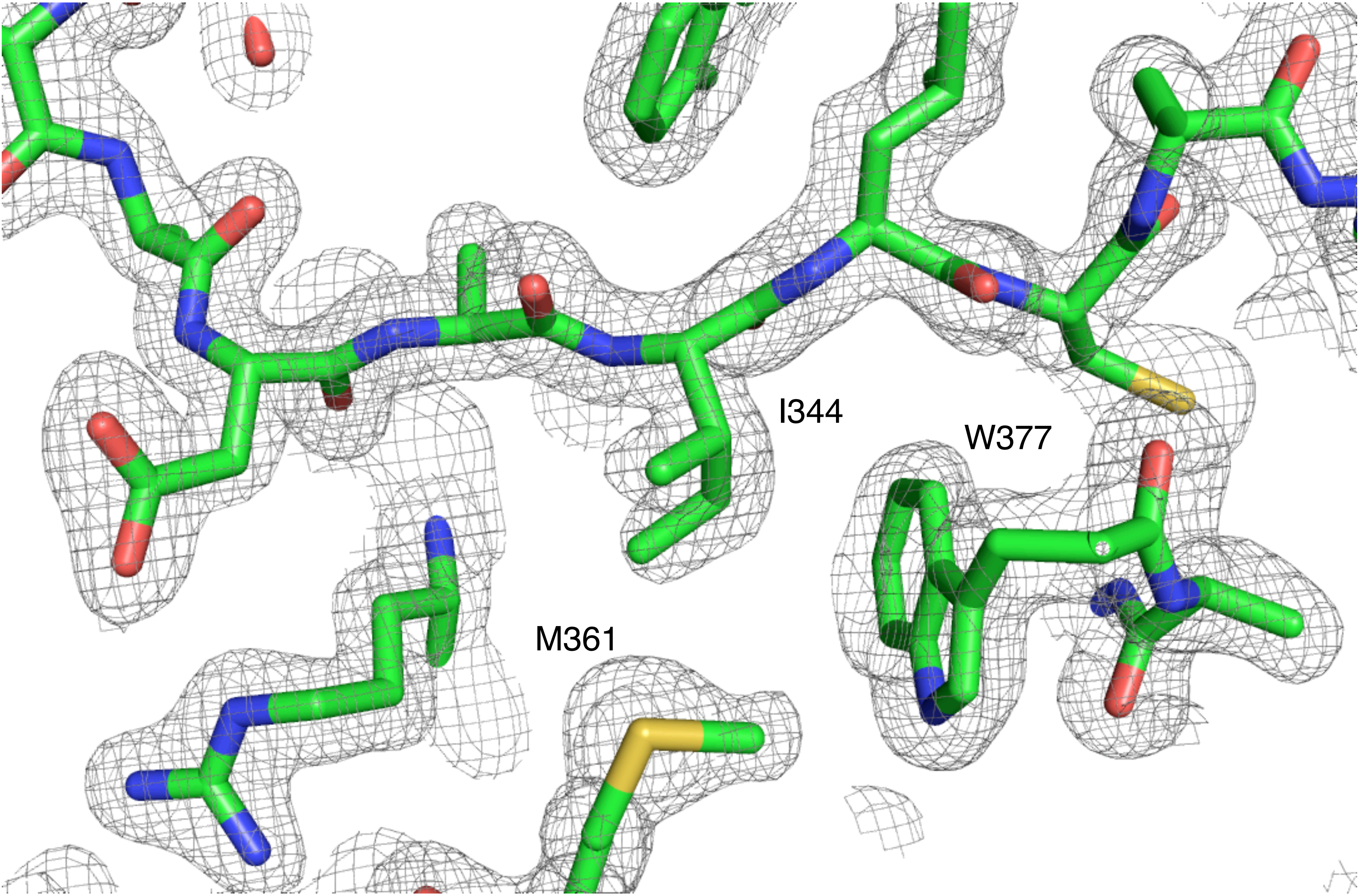

Supplementary Figure 4 Miller et al.
